# Social Contexts and Immigration Policies Directly Impact Immigrant Health

**DOI:** 10.3390/ijerph22040586

**Published:** 2025-04-09

**Authors:** Maria De Jesus, Ernesto Castañeda

**Affiliations:** 1 Department of Environment, Development, & Health, School of International Service, American University, Washington, DC 20016, USA; 2 The Immigration Lab, Center for Latin American and Latino Studies, American University, Washington, DC 20016, USA; 3 Department of Sociology, College of Arts and Sciences, American University, Washington, DC 20016, USA

At the end of 2023, an estimated 117.3 million people worldwide were forcibly displaced due to persecution, conflict, violence, or human rights violations [1]. This figure translates to 1 in every 69 people worldwide being forcibly displaced or 1.4% of the global population [1]. The figure includes internally displaced persons (68.3 million), refugees under the mandate of the United Nations High Commissioner for Refugees (UNHCR) (31.6 million), asylum seekers (6.9 million), refugees under the mandate of the United Nations Relief and Works Agency for Palestine Refugees in the Near East (6 million), as well as others in need of international protection (5.8 million) [2]. Low- and middle-income countries hosted 75 per cent of the world’s refugees and other people in need of international protection [2]. International migration continues to be a major driver of economic growth and a key contributor to these countries’ GDPs [2,3].

Global shifts in migration patterns, climate change, rising health and social inequities, and environments that are marked by poverty and violence significantly affect the health of migrants. In these “contexts of vulnerability”, migrants often face a range of physical and mental health challenges before, during, and after their migration [4,5,6]. They are also at heightened risk of isolation, exclusion, discrimination, and insecurity, all of which further harm their well-being [7]. Migrants in irregular or undocumented situations face even greater risks, including exploitation, trafficking, detention, and deportation [8,9]. When evaluating the health and well-being of migrants worldwide, it becomes evident how social determinants of health, social structures, and multi-sectoral policies play a central role [10,11].

The experience of migration and migration status are key social determinants of health and well-being, as they place individuals in circumstances that can significantly affect their health [11,12]. The conditions surrounding migration and its health impacts are shaped by a range of factors that differ across migrant groups and legal statuses [13,14,15]. The social determinants of health (SDH) framework, highlights the critical role of social, economic, and political factors, along with structural mechanisms, in influencing individual health behaviors and risks (Figure 1) [16].

Key SDHs include migration and migration status, quality childcare and education, employment status, gender equality, income, and access to adequate housing, nutritious foods, and healthcare [17]. The upstream and intermediary determinants help explain why there are differential risks for disease and health outcomes across populations [18,19]. They also create social stratification and health inequities based on migration status, income, education, class, gender, and race/ethnicity [17,20,21].

This Special Issue, titled “Migration and Migration Status as Key Determinants of Health”, explores five key themes, which are outlined in detail below.

## 1. Migration, Mental Health, and Access to Care

The first set of articles share a common focus on how immigration-related stress, discrimination, and systemic barriers contribute to mental health challenges among migrant populations, while also highlighting the complexities of care access and the coping strategies that migrants use to navigate their experiences. In the article “Investigating the Potential Double-Edged Score of Immigration-Related Stress, Discrimination, and Mental Health Access”, Andrews et al. explore how discrimination and immigration-related stress lead to post-traumatic stress disorder and depression among Latinx migrants and, in turn, also indirectly encourage help-seeking behaviors, especially for those dealing with trauma. Similarly, Espinoza-Kulick and Cerdeña elucidate the systemic barriers that Latinx migrants face in accessing mental health services in their article titled “We Need Health for All: Mental Health and Barriers to Care among Latinx in California and Connecticut”. The authors elucidate the systemic barriers that Latinx migrants face in accessing mental health services. The authors emphasize the need for culturally competent mental health services that account for language barriers, gendered experiences, and the intersecting challenges of immigration status and socioeconomic insecurity.

In the article “Hoping for a Better Future during COVID-19: How Migration Plans are Protective of Depressive Symptoms for Haitian Migrants Living in Chile”, Chen et al. demonstrate that Haitian migrants in Chile experienced high levels of depression, exacerbated by changes in migration plans due to the pandemic. Their study reveals that planning to leave Chile was a protective factor against Haitians’ depressive symptoms, suggesting that hope and future aspirations can act as crucial coping mechanisms for migrants struggling with mental health, even in the face of discrimination and uncertainty. Similarly, De Jesus et al.’s article, “‘Living in Confinement, Stopped in Time’: Migrant Social Vulnerability, Coping and Health during the COVID-19 Pandemic Lockdown in France”, examines the compounded health, protection, and socioeconomic crises faced by asylum seekers and undocumented migrants during the COVID-19 lockdown in France. The study reveals how lockdown measures exacerbated existing vulnerabilities and emphasizes the need for inclusive health policies that address the root causes of health inequities. The authors advocate for a holistic, migrant-inclusive approach to well-being.

Together, these articles illustrate the significant mental health challenges that are faced by migrants, including discrimination, trauma, and a lack of access to mental healthcare. They underscore the need for policies and services that address these barriers, foster resilience, and support mental health through both practical and emotional coping strategies, while also recognizing the critical role of hope and future planning in the mental well-being of migrant communities.

## 2. Migrant Integration and Resettlement: Challenges and Nuances

A second theme emerges around the challenges and nuances of migrant integration and resettlement, particularly the impacts of social connection, forming identity, and overcoming barriers to economic and social stability. In the article “Reskilled and Integrated, but How? Navigating Trauma and Temporary Hardships”, Aydiner and Rider explore how highly educated Turkish migrants, forced to leave their home country due to political upheaval, face the complex task of transferring their professional credentials and re-establishing their economic status in the United States. While their economic situation eventually improves, the process of re-education and credential recognition remains time-consuming and challenging, affecting both their economic well-being and mental health. In the article titled “‘Step by Step We Were Okay Now’: An Exploration of the Impact of Social Connectedness on the Well-Being of Congolese and Iraqi Refugee Women Resettled in the United States”, Bletscher and Spiers investigate the gendered impacts of resettlement on Congolese and Iraqi refugee women in the U.S. The study highlights how these women struggle with building social networks that provide essential resources and emotional support.

In their article “A Qualitative Study of Adolescents from Refugee Backgrounds Living in Australia: Identity and Resettlement”, Khawaja and Schweitzer delve into the identity formation of refugee youth and the challenges of navigating adolescence while integrating into a new cultural environment in Australia. The study highlights the importance of fostering a sense of belonging and the role of school in helping these young people build resilience and a cohesive sense of self, despite the disruptions caused by displacement. In the article “Do Resettled People Adapt to their Current Geographical Environment? Evidence from Poverty-Stricken Areas of Northwest Yunnan Province, China”, Qu et al. examine how resettled populations in China adapt to their new environments following poverty alleviation policies. The study finds that while resettlement improves living conditions, barriers to environmental adaptation and the risk of returning to poverty persist. The authors emphasize the need for policies focusing on employment, public services, and social safety nets to better support the long-term integration of resettled populations.

These articles underscore the multifaceted process of the integration and resettlement of migrants, with a focus on the significant challenges that migrants face in terms of economic stability, identity reconstruction, and adapting to new environments. A central takeaway is the critical role of supportive policies in fostering successful resettlement.

## 3. The Role of Social Networks and Connections in Shaping Migrants’ Health and Well-Being

A common thread across the next set of articles is the importance of social networks and connections in shaping the experiences and well-being of migrants. These networks—whether familial, cultural, or community-based—play a significant role in helping migrants navigate challenges relating to identity, health, and integration. In Koku’s article, “The Effect of Stigma and Social Networks on Role Expectations among African Immigrants Living with HIV”, we learn how African immigrants living with HIV use their social networks to negotiate stigma and role expectations. The author shows how these social networks, embedded in both home and host countries, influence health outcomes, identity reconstruction, and the ability to cope with migration stressors. Wang and Cao’s article, “Network Diversity and Health Change among International Migrants in China: Evidence from Foreigners in Changchun”, explores the dual social networks that migrants form—connections to both their home and host country.

In the article “Leaving the Homestead: Examining the Role of Relative Deprivation, Social Trust, and Urban Integration among Rural Farmers in China” by Si, Jiang, and Meng, the authors demonstrate how social networks influence rural-to-urban migration decisions. The authors underscore how social deprivation, economic hardship, and emotional isolation affect the willingness of rural farmers to leave their homesteads, with social trust acting as a crucial factor in this decision-making process. Lastly, in the article “A Systematic Review of Evidence-Based Family Interventions for Trauma-Affected Refugees”, Mak and Wieling highlight the centrality of family connections for refugees from collectivistic cultures. The authors suggest that effective interventions for trauma-affected refugee families must take the relational and cultural dynamics within these family networks into account to improve mental health outcomes and ensure long-term well-being.

In summary, these articles demonstrate that social networks—whether family, cultural ties, or community connections—are essential for migrants’ well-being. They help migrants cope with health challenges, navigate acculturation stress, and make critical decisions about their lives in both host and home countries. These networks also shape how migrants perceive their identity and role within society, influencing their health outcomes and integration into new environments.

## 4. The COVID-19 Pandemic: Migration, Health Inequities, and Structural Factors

The COVID-19 pandemic exacerbated existing health inequities among migrant populations, with structural and policy-driven factors significantly impacting their health outcomes. Across the next set of studies, an overarching theme is the disproportionate vulnerability of migrants to both the virus and its social and economic consequences, often compounded by exclusionary policies and systemic inequities. In the article “The Implications of Health Disparities: A COVID-19 Risk Assessment of the Hispanic Community in El Paso”, Cione et al. explore how pre-existing inequities, such as high rates of chronic conditions and socioeconomic factors, made Hispanic communities in El Paso more susceptible to severe COVID-19 outcomes. The authors underscore the importance of addressing structural inequality to prevent future health crises and the importance of large-scale surveys, including the collection of social and health data, to have a baseline for evaluating the strengths, challenges, and vulnerabilities of a community’s health.

In the article “Movement Pandemic Adaptability: Health Inequity and Advocacy among Latinx Immigrant and Indigenous Peoples”, Viveros Espinoza-Kulick highlights how immigrant and Indigenous communities, despite facing systemic barriers such as immigration status and language challenges, have relied on mutual aid and advocacy networks to provide culturally relevant resources. The paper illustrates the importance of community solidarity in mitigating the impact of health inequities during the pandemic.

In the article “The Most Vulnerable Hispanic Immigrants in New York City: Structural Racism and Gendered Differences in COVID-19 Deaths”, Fuentes-Mayorga and Cuecuecha Mendoza demonstrate how structural racism and gender disparities shaped the COVID-19 mortality rates among Hispanic immigrants. The study reveals how Hispanic men, especially those working in essential, high-risk jobs, faced higher mortality risks, while gendered experiences and spatial segregation further exacerbated the inequities. Similarly, in the article “COVID-19 and Policy-Induced Inequalities: Exploring How Social and Economic Exclusions Impact ‘Temporary’ Migrant Men’s Health and Wellbeing in Australia”, Rung examines how Australia’s COVID-19 response policies, which excluded men who had migrated temporarily and their families from economic support, exacerbated the health and well-being challenges for this group. The study reveals the role of “sub-citizenship” status, where temporary migrants face heightened vulnerability to both the virus and the socioeconomic consequences of exclusionary policies.

These articles reveal how migration status, social exclusion, and structural racism intertwine to amplify the impact of the pandemic on migrant communities. They emphasize the urgent need for inclusive policies that address both health inequities and the socioeconomic vulnerabilities that migrants face, while also fostering community-led solutions for resilience.

## 5. Migrants and Experiences of Violence

A central theme across the next four studies is the complex and violent experiences that are faced by many migrants, shaped by both structural and interpersonal factors. Migrants navigate multiple layers of violence, from the physical dangers of crossing borders to the emotional and physical toll of separation and the psychological impact of hostile media narratives.

In the article by Crocker et al., titled “‘Es Muy Tranquilo Aquí’: Perceptions of Safety and Calm among Binationally Mobile Mexican Immigrants in a Rural Border Community”, Mexican immigrants’ perceptions of community safety are contrasted with the violence that they associate with conditions across the border. While some migrants feel safe in their rural communities, they remain deeply aware of the violence in neighboring regions, which informs their overall sense of security and health. In Piñones-Rivera et al.’s article “Indigenous Border Migrants and (Im)Mobility Policies in Chile in Times of COVID-19”, the focus shifts to the structural violence that is embedded in immigration and healthcare policies. Indigenous migrants in Chile face violence not only from border closures and healthcare inaccessibility but also from criminalization by state actors. These migrants must navigate dangerous routes for basic healthcare, exposing them to exploitation and abuse.

In the article “‘Mi Corazon se Partió en Dos’: Transnational Motherhood at the Intersection of Migration and Violence”, Cook Heffron, Wachter, and Rubalcava Hernandez delve into how gender-based violence affects Central American women’s decisions to migrate, often forcing them to leave their children behind. The violence that they experience, from economic insecurity to sexual violence, shapes their understanding of motherhood and underscores the emotional costs of migration. In the article “The Influence of Media Coverage on the Negative Perception of Migrants in Chile”, Scherman et al. demonstrate how the media’s portrayal of migrants influences public opinion, fostering xenophobia and discrimination. Negative media coverage exacerbates the social violence that is experienced by migrants, turning public perception against them and heightening the risks that they face in their host country.

Together, these articles highlight how migrants face multiple forms of violence—physical, structural, emotional, and social. These experiences are compounded by migration policies and media portrayals that shape the environments in which migrants live, work, and raise families. The violence that they endure is not just a result of external threats but also deeply intertwined with the social and political systems which they navigate.

## 6. Conclusions

This volume underlines key themes at the intersections of health, migration, and migration status. Firstly, it examines the significant mental health challenges that are faced by migrants, particularly relating to discrimination, trauma, and limited access to care. Secondly, it underscores the multifaceted process of migrant integration, focusing on the substantial hurdles that migrants encounter in terms of economic stability, identity reconstruction, and adapting to their new environment. Thirdly, it demonstrates how social networks and connectedness influence migrants’ perceptions of their identity and role within society, ultimately shaping their health outcomes and integration experiences. Fourthly, it reveals how migration status, social exclusion, and structural racism amplified the impact of the pandemic on migrant communities. Lastly, it explores how migrants’ experiences with different forms of violence—physical, structural, emotional, and social—are further exacerbated by migration policies and media portrayals that influence their social contexts.

Migration and migration status are key determinants of health. The 2030 Sustainable Development Agenda, the Global Compact for Safe, Orderly, and Regular Migration and other international frameworks highlight the critical need to monitor health data at the migration–health nexus [22]. These initiatives are essential for tracking progress towards ensuring that no one is left behind, especially in achieving health-related goals and targets that promote equity for all, regardless of migration status. This work calls for a comprehensive, in-depth understanding of the social determinants of health that affect migrants, advocating for inclusive policies and practices that guarantee equitable access to healthcare and protection of migrants’ rights, health, and well-being, regardless of their migration status.

## Figures and Tables

**Figure 1:**
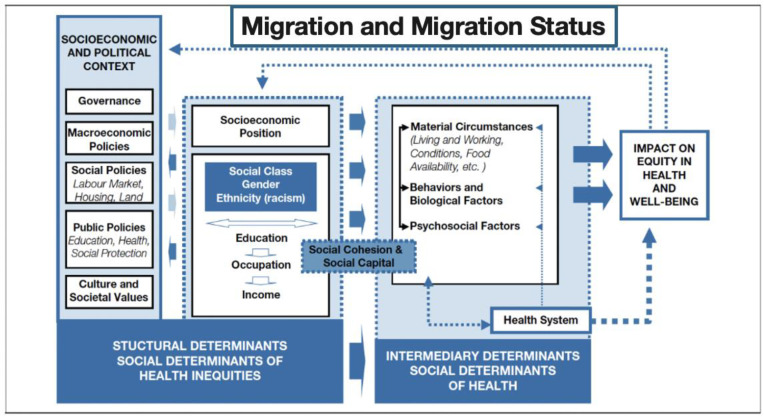
A conceptual framework for the social determinants of health. World Health Organization, 2010.
